# Identification of DNA methylation changes associated with human gastric cancer

**DOI:** 10.1186/1755-8794-4-82

**Published:** 2011-12-02

**Authors:** Jung-Hoon Park, Jinah Park, Jung Kyoon Choi, Jaemyun Lyu, Min-Gyun Bae, Young-Gun Lee, Jae-Bum Bae, Dong Yoon Park, Han-Kwang Yang, Tae-You Kim, Young-Joon Kim

**Affiliations:** 1 Department of Biochemistry, College of Life Science and Technology, Yonsei University, Seoul, Korea; 2 Department of Integrated Omics for Biomedical Science, WCU Program of Graduate School, Yonsei University, Seoul, Korea; 3 Cancer Research Institute, Department of Internal Medicine, Seoul National University College of Medicine, Seoul, Korea; 4 Department of Bio and Brain Engineering, KAIST, Daejeon, Korea; 5 Computational and Systems Biology, Genome Institute of Singapore, Singapore, Singapore

## Abstract

**Background:**

Epigenetic alteration of gene expression is a common event in human cancer. DNA methylation is a well-known epigenetic process, but verifying the exact nature of epigenetic changes associated with cancer remains difficult.

**Methods:**

We profiled the methylome of human gastric cancer tissue at 50-bp resolution using a methylated DNA enrichment technique (methylated CpG island recovery assay) in combination with a genome analyzer and a new normalization algorithm.

**Results:**

We were able to gain a comprehensive view of promoters with various CpG densities, including CpG Islands (CGIs), transcript bodies, and various repeat classes. We found that gastric cancer was associated with hypermethylation of 5' CGIs and the 5'-end of coding exons as well as hypomethylation of repeat elements, such as short interspersed nuclear elements and the composite element SVA. Hypermethylation of 5' CGIs was significantly correlated with downregulation of associated genes, such as those in the *HOX *and histone gene families. We also discovered long-range epigenetic silencing (LRES) regions in gastric cancer tissue and identified several hypermethylated genes (*MDM2*, *DYRK2*, and *LYZ*) within these regions. The methylation status of CGIs and gene annotation elements in metastatic lymph nodes was intermediate between normal and cancerous tissue, indicating that methylation of specific genes is gradually increased in cancerous tissue.

**Conclusions:**

Our findings will provide valuable data for future analysis of CpG methylation patterns, useful markers for the diagnosis of stomach cancer, as well as a new analysis method for clinical epigenomics investigations.

## Background

Gastric cancer is the second leading cause of cancer deaths worldwide after lung cancer, resulting in more than 800,000 deaths worldwide every year [1]. The current 5-year survival rate of individuals diagnosed with gastric cancer is only 20-30%, with this low rate being attributable to the fact that most cases are already in an advanced stage when diagnosed. As in all cancers, early detection remains the most promising approach for improving the survival rate. Hence, understanding the cause of tumorigenesis in human gastric tissue is essential.

Infection with *H. pylori *is a well-established and common cause of gastric cancer. However, alterations in various genetic factors are also important in increasing gastric cancer risk. It is well known that chromosomal instability originating from genetic factors such as microsatellite instability as well as *KRAS *and *p53 *mutations result in the development of tumors. Several genomic studies have identified germline mutations in specific genes [2-4] and disease susceptible loci [5,6] for gastric cancer. Recent studies comparing gastric cancer and normal tissue have identified a number of genetic markers, including diagnostic markers [*NF2*[7], *INHBA*[8], *SFRP4*[9]], prognostic markers [*CD9*[10], *CDH17*[11], *PDCD6*[12]], and gastric cancer-associated genes [*MUC13*[9], *CLDN1*[13], *Ki67 *and *CD34*[14]]. In addition, epigenetic mechanisms such as DNA methylation and histone modifications have been found to be important in regulating the expression of genes involved in the biology and disease of the gastrointestinal tract [15].

DNA methylation plays an essential role in eukaryotes and is associated with a number of key mechanisms including genomic imprinting, X chromosome inactivation, aging, and carcinogenesis. Alteration of DNA methylation in the genome is found in almost all types of cancer and can lead to changes in gene expression, such as over-expression of oncogenes and silencing of tumor suppressor genes during cancer development [16]. Several studies have shown that accumulation of genetic and epigenetic alterations in gastric precancerous lesions may affect a large number of targets, such as DNA repair system components, tumor suppressors, oncogenes, cell cycle regulators, growth factors, and adhesion molecules [17-20]. However, these studies have been primarily focused on a few candidate genes or covered only a portion of the whole genome. Thus, accessing a global view of the epigenetic changes associated with cancer development has been difficult. In particular, understanding DNA methylation changes in the intragenic regions, CpG islands, intergenic regions, and repeat sequences remains limited. Consequently, there is great interest in genome-wide analysis of aberrant DNA methylation in these regions.

For comprehensive genome-scale profiling of DNA methylation in embryogenesis and carcinogenesis, high-resolution whole genome sequencing methods such as BS-seq [21-24], MeDIP-seq [25,26], and MethylCap-seq [27-29] have been developed. Despite the rapid development of sequencing-based mapping technology, there is still a lack of comparative research, which is critical for clinical epigenomics studies, including those focused on cancer. Unlike microarray-based approaches, sequencing data are produced in a format that is not amenable to differential analysis, and the analysis workflow has not been standardized. Hence, computationally inexpensive normalization methods are needed to handle the computational burden of processing large-size, high-resolution sequencing data.

Here, we introduced a normalization algorithm, which takes into account the sample-specific total read density, the spatial distribution of CpG loci, and background sequencing bias. We then created a comprehensive whole-genome methylome of normal gastric tissue, gastric cancer tissue, and metastatic lymph nodes using the MethylCap-seq method and obtained detailed information on its perturbation during carcinogenesis and metastasis. This is readily applicable to a comparative analysis of methylomes and other types of epigenomic data, and it has particular implications for clinical epigenomics.

## Methods

### Gastric tissue samples

We obtained three snap-frozen gastric tumors and matched normal gastric tissue from Seoul National University College of Medicine for methylome study. Additionally, twenty-eight matched pairs of normal and tumor stomach tissues were obtained for further confirmation. All samples were obtained by endoscopic resection during examination of the patients who gave informed consent.

### Methylated DNA recovery assay (MIRA)

Genomic DNA from 25 mg of gastric tissue was purified by using DNeasy Blood & Tissue Kit (Qiagen, Valencia, CA). Genomic DNA samples from 3 individuals were pooled at the same concentration. MIRA was carried out as previously described [30-32]. Briefly, GST-tagged MBD2b and His-tagged MBD3L1 proteins were prepared as described. 15 ug of genomic DNA was fragmented to 100 ~ 500 bp by sonication and incubated with 28 ug of purified GST-MBD2b protein, 28 ug of His-MBD3L1 protein and 7 ug of JM110 Bacterial DNA for 6 hours. 30 ul of MagneGST beads (Promega, Madison, WI) preblocked with 7 ug of JM110 bacterial RNA were added and incubated at 4°C with rotating for 45 minutes in final 600 ul of MIRA binding reaction mixture. Beads were washed three times with 1 ml of washing buffer, and methylated fragments were eluted by incubation at RT for 5 minutes and then 56°C for 30 minutes with 30 ul of TE containing RNase A (100 ug, Qiagen) and Proteinase K (15 ug, Qiagen). Eluted DNA fragments were further purified by using Qiaquick PCR purification kits (Qiagen).

### Illumina Genome Analyzer sequencing

We used 10 ng of eluted DNA for Illumina Genome Analyzer sequencing. Following ligation of a pair of Solexa adaptors, ligation products with the maximum insert size of 200 bp were gel purified on 2% agarose and subjected to PCR amplification. Cluster generation and 36 cycles of sequencing were performed following the manufacturer's instructions. We sequenced 120 ul of adaptor-ligated, size-fractionated DNA (2 ~ 4 pM) on the Illumina Genome Analyzer. Sequence tags were mapped to the human genome (UCSC hg18 database based on NCBI Build 36.1 assembly) using the Solexa Analysis Pipeline (version 0.3.0). Sequenced reads of 34 bp (excluding the first and last nucleotide) that passed quality control filters were used.

### Data processing and MES calculation

We extended the 3' end of the 34-bp reads by 200 bp to cover DNA fragments bound by the MBD proteins. The readout was converted to browser extensible data (BED) files for visualization in the UCSC genome browser http://genome.ucsc.edu/. We counted overlapping sequence tags at 50 bp resolution. To find enriched genomic regions, the number of mapped reads in a sliding window of 1 kb was compared to the total number of reads or the background number of reads in the genome. As such, MES was calculated in two ways; one is as the log2 of (target read count/target size)/(total read count/genome size) and floored to zero, the other is as the log2 of (target read count/target size)/(background read count/background size) and floored to zero. To adjust for background sequencing bias, MESbg was calculated in the same manner for input sequencing without affinity purification and subtracted from MES.

### Genomic positions of CGIs, promoters, transcript bodies, CDSs, and repetitive elements

All genomic positions of CGIs, transcripts and repeat elements were downloaded from the UCSC genome browser. A total of 27,639 CGIs (except randomly located CGIs) were predicted by the following criteria: GC content of 50% or greater, length greater than 200 bp, and ratio greater than 0.6 of observed number of CpG dinucleotides to the expected number [33]. The NCBI mRNA reference sequences collection (RefSeq from release version 46; March 11, 2011) was used for identifying transcription units with the defined transcription start, end sites and CDS start, end sites. For promoters, we used the region 500 bp upstream ~ 500 bp downstream of the transcription start site. We obtained ~ 5 million repeat locations that had been determined by the RepeatMasker program based on the RepBase library of repeats.

### Methylation level of genomic elements

The methylation level of a CGI, promoter, gene-body, and repeat element was estimated by means of MES overlapping each element. MES = 0 was used to define unmethylated elements. To measure hypermethylation or hypomethylation in cancer, we calculated the differential MESs as (Cancer MES - Normal MES). Differential MES > 1.0 was used as a threshold. To understand the functions of selected genes, we used the ontology classification of genes through the DAVID Functional Annotation Clustering tool http://david.abcc.ncifcrf.gov/.

### Gene expression analysis

The microarray product used in this study was Codelink Human Whole Genome 55 K chip (GE Healthcare, USA). All experimental procedures including cRNA target preparation, hybridization, post-hybridization dye coupling were performed using vendor recommended protocols. The result files were imported into GeneSpring GX 7.3 (Agilent Technologies, USA) for filtering and basic statistical analysis. Among 55 K genes on the microarray, only the genes with present flags in at least 50% of samples were selected for subsequent analysis. The microarray data were deposited at the GEO http://www.ncbi.nlm.nih.gov/geo/ (accession number GSE33651).

### MIRA and real-time qPCR

MIRA was performed on four additional individual samples. DNA was purified from the supernatant and monitored by real-time qPCR using Roche 480 machine. The sequences of used primers are presented in Additional file 1: Table S1.

### Bisulfite treatment, methylation-specific PCR and pyrosequencing

We isolated the genomic DNA from individual sample by using a Qiagen DNeasy Tissue Kit (Qiagen). Bisulfite treatment was carried out using the EZ DNA methylation gold kit (Zymo research) according to the manufacturer's instructions. Bisulfite-treated DNA was stored at -80°C until further use. The primers used for MSP were designed using Methprimer [34], and are shown in Additional file 1: Table S1. PCR was performed with HotStarTaq Polymerase (Qiagen) and included an initial incubation at 95°C for 15 min, followed by 40 cycles of 95°C for 1 min, 59°C for 1 min and 72°C for 40 sec, followed by one cycle of 72°C for 10 minutes. MSP products were separated on 2% agarose gels and visualized by ETBR staining. The pyrosequencing reactions were automatically performed with a PSQ 96 system (Pyrosequencing AB) according to the manufacturer's instructions. Briefly, the biotinylated PCR product (50 ul) was purified by using streptavidin-sepharose beads (Amersham Biosciences). The purified product was loaded into the reagent cartridge with the enzyme, substrate and dNTP included in the PSQ96 SNP Reagent Kit (Pyrosequencing AB). The sequencing primers for pyrosequencing are shown in Additional file 1: Table S1.

## Results

### Processing of MIRA-seq methylome data

We purified the methylated DNA enriched through MIRA (methylated CpG island recovery assay) and sequenced the DNA using next-generation sequencing. DNA methylation levels were determined using sequencing read counts of the corresponding regions, at 50 bp intervals, as described under Methods. We created DNA methylation maps for both normal and cancerous gastric tissues. For each sample, we obtained about 10 million sequence reads (Additional file 1: Table S2). Each methylome contained ~140 million CpG reads, covering ~48% of all genomic CpG sites excluding centromeres (Additional file 1: Table S3). The average coverage of CpG reads in each methylome was 4.5X. In support of the high sensitivity of MIRA, genomic segments containing only one CpG had higher read counts than those with no CpG (*p *value = 0), suggesting that single CpG changes could be resolved using MIRA. The average sequence reads increased in proportion to the number of CpGs within a 50-bp interval, and in fact, MIRA coverage was not low, even for regions of low CpG density (Additional file 2: Figure S1). Taken together, these results show that MIRA was successful in recovering a sufficient fraction of methylated regions. As for the accuracy of MIRA, ~99% of MIRA-captured fragments had at least one CpG site within their sequence, indicating a low false detection rate.

To measure enrichment of local methylation signals, we calculated methylation enrichment scores (MESs) by obtaining a read count in a given region and then performing normalization to control for the total read count (MESt) in the sample (global normalization) or the local read count (MESl) in a user-defined surrounding region (local normalization) (see Methods). This enables a direct comparison of independent samples with different read density. We then carried out a logarithmic transformation of the derived score. Along with having other mathematical merits, this provides the benefit of variance stabilization, particularly for high read counts, which are often coupled with high technical variations that may introduce significant bias in the data.

We assessed the statistical significance of the MES in two ways. Randomized MESs were generated numerically by permuting the genomic positions of our sequence reads. The background MES (MESbg) was experimentally obtained by sequencing the normal genome without affinity purification. As expected, the real data yielded markedly higher enrichment scores (Additional file 2: Figure S2). Notably, MESbg was higher than MES from randomized genomes, an indication that background sequences alone can create enrichment, probably due to chromatin accessibility and amplification bias. Consistent with recent reports [35], this illustrates the need for a proper calibration for inherent sequencing bias. Therefore, we normalized our MES with MESbg.

To find the optimal condition for normalization, we compared the statistical fitness of various normalization methods. Tag distribution along the genome can be modeled by the Poisson distribution [36,37]. The goodness of fit was tested using the Kolmogorov-Smirnov test. In this test, a low D statistic indicates a good fit. While the Poisson model outperformed the Gaussian overall, the MES showed a better fit than raw read counts (Additional file 2: Figure S3), illustrating the rare event nature of the log-scaled read count measure. The normalized MESl calibrated by control sequencing (MESbg) yielded even better results than normalized MESt calibrated by control sequencing (MESbg).

### Global and chromosomal views of DNA methylation

**Figure 1:**
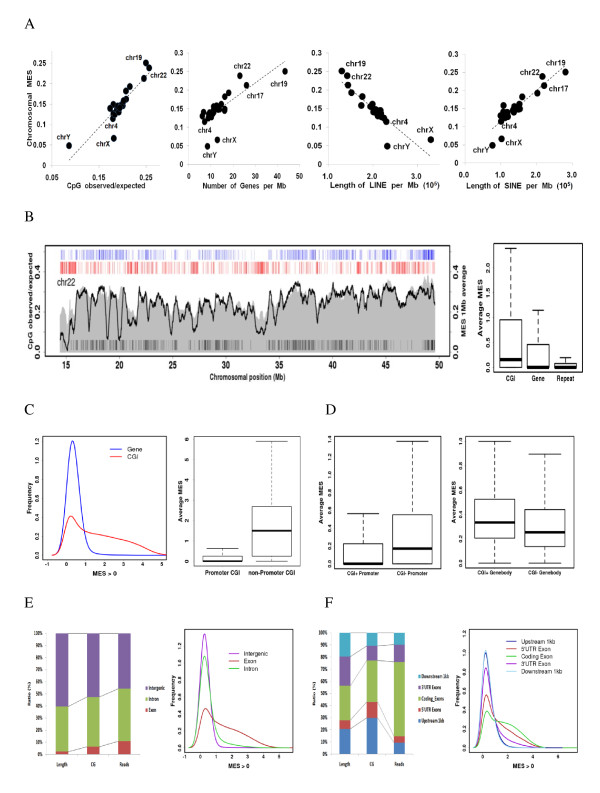
**Methylation patterns of normal gastric tissue**. (A) Chromosome-wide average MES is shown as a function of the average CpG density, gene density (the number of genes per Mb), LINE quantity (the length of LINE per Mb), and SINE quantity (the length of SINE per Mb) for each chromosome. (B) For chromosome 22, the average CpG density (shaded gray) and MES (black curve) were obtained in 1-Mb sliding windows. The positions of transcribed genes (black bars at the bottom), CG islands (blue bars at the top), and long repeats (> 1 kb; red bars on the top) are compared against the backdrop of DNA methylation and CpG density (left). The average MES for CGIs, gene bodies, and repeats (right). (C) The distribution of gene bodies and CGI MES (left). The average MES for promoter-associated and promoter-independent CGIs is shown to the right. (D) The average MES for promoter subgroups, based on the existence of CGI (left). (E) Basic information on intergenic, exonic, and intronic regions, according to length, CpG number, and mapped reads (left). The distribution of intergenic, exonic, and intronic MESs is shown to the right. (F) Basic information on the upstream 1-kb region, 5' UTR exons, coding exons, 3' UTR exons, and downstream 1-kb region according to length, CpG number, and mapped reads (left). The distribution of the MES for each element is shown to the right.

After confirming the best method for scoring genome-wide methylation levels at 50-bp intervals, we first examined the chromosomal methylation patterns of normal samples. The average MES calculated for each chromosome suggested that CpG-rich and gene-rich chromosomes tend to be highly methylated (Figure 1A). The methylation levels of chromosomes with large amounts of long interspersed nuclear elements (LINEs) were relatively low (e.g., chromosome 4). Interestingly, the quantity of short interspersed nuclear elements (SINEs) was proportional to the chromosome methylation pattern. This is likely caused by the fact that SINEs are typically clustered in gene-rich regions. Sex chromosomes were globally hypomethylated with lower CpG density and higher repeat content than autosomes. Since we used the tissues taken from a male in this experiment, the global hypomethylation of the X chromosome observed is not associated with X inactivation. Chromosome-wide views recapitulated high CpG density and high methylation around gene-rich (see black bars at the bottom) and CGI-rich regions (see blue bars at the top) (Figure 1B). In contrast, low CpG density and low methylation were observed around gene-poor regions that were rich in long-range repeats (> 1 kb) (see red bars at the top). The average MES suggests that the methylation level of CGIs is considerably higher than that of genic regions or repeats (Figure 1B).

Generally, CGIs tend to remain methylation free in normal tissue. To analyze the high methylation patterns of CGIs, we checked the average MES distribution and found a slightly bimodal pattern (Figure 1C). About 66% (11,376/17,284) of CGIs in the left peak overlapped with a promoter (1 kb by our definition). In contrast, 13% (1,386/10,357) of CGIs in the right peak overlapped with a promoter, suggesting that most promoter-associated CGIs are unmethylated. In contrast to promoter-related CGIs, promoter-independent CGIs were heavily methylated (Figure 1C). Although most CGI-positive promoters were not methylated, CGI-negative promoters showed relatively high methylation levels (Figure 1D). We also checked the methylation level of promoters by CpG density as previously defined [38] (Additional file 3: Table S4). The methylation pattern of promoters was inversely related to CpG density (Additional file 2: Figure S4). On the other hand, CGI-containing gene bodies had higher methylation levels than those without CGIs (Figure 1D).

Next, we analyzed methylation enrichment patterns at various annotated genomic elements to explore regions that were preferentially methylated. Genic regions occupy about 40% of human genome, but about 53% of the total reads is within this region, with the majority of reads being located in the intronic region (Table 1). Although a significant proportion of methylated fragments fall within intronic regions, the ratio of mapped reads to the length of exons is considerably higher than that for introns, suggesting that exons are more highly methylated than introns (Figure 1E). Within gene-associated regions, the enrichment of coding exons is even higher than that of other regions as previously reported (Figure 1F and Table 2) [39]. This strongly suggests that methylation plays a role in exon regulation.

**Table 1:** Human genome and normal sample information of genic and intergenic region

Human Genome Information					Normal Sample Information		Relative Enrichment Ratio	
**Functional Category**	**Length (bp)**	**Ratio**	**# of CpG**	**Ratio**	**Reads**	**Ratio**	**vs. length**	**vs. CpG Count**

Genic	1,184,139,094	39.46	13,262,253	47.09	20,854,434	53.25	1.35	1.13

Exon	68,035,894	2.27	1,808,089	6.42	4,350,405	11.11	4.90	1.73

Intron	1,122,817,725	37.41	11,613,113	41.23	17,358,273	44.32	1.18	1.07

Intergenic	1,816,976,186	60.54	14,901,610	52.91	18,310,273	46.75	0.77	0.88

Human Genome	3,001,115,280	100	28,163,863	100	39,164,707	100	1.00	1.00

**Table 2:** Human genome and normal sample information of gene annotated regions

Human Genome Information					Normal Sample Information		Relative Enrichment Ratio	
**Functional Category**	**Length (bp)**	**Ratio**	**# of CpG**	**Ratio**	**Reads**	**Ratio**	**vs. length**	**vs. CpG Count**

Upstream 1 kb	24,468,069	0.82	937,748	3.33	535,593	1.37	1.68	0.41

5'UTR Exons	8,436,529	0.28	411,563	1.46	292,654	0.75	2.66	0.51

Coding Exons	33,384,619	1.11	1,077,913	3.83	3,448,755	8.81	7.92	2.30

3'UTR Exons	28,387,978	0.95	378,012	1.34	806,832	2.06	2.18	1.53

Downstream 1 kb	23,136,263	0.77	340,866	1.21	551,071	1.41	1.83	1.16

Human Genome	3,001,115,280	100	28,163,863	100	39,164,707	100	1.00	1.00

### Changes in DNA methylation patterns associated with gastric cancer

**Figure 2:**
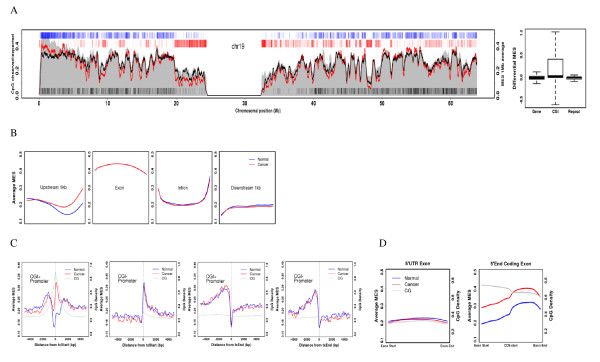
**Comparison of methylation patterns in normal and cancerous tissue**. (A) Average MES curve for normal (black) and cancerous (red) tissue in chromosome 19 (left). The average MES for CGIs, gene bodies, and repeats (right).(B) DNA methylation of gene annotated elements. Each element (upstream 1 kb, exon, intron, and downstream 1 kb) were partitioned into 20 bins and the average MES was obtained for each bin of all corresponding elements. (C) DNA methylation at transcript ends and coding region ends. The average MES was obtained in a sliding 50-bp window according to its distance from the transcript start (first) and end (second) for CGI-positive promoters as well as the transcript start (third) and end (fourth) for CGI-positive promoters. (D) DNA methylation of total 5' UTR exons (left) and 5' UTR coding exons (right).

When the average chromosomal MES of the cancer methylome was compared to that of control tissue, we found that all chromosomes in the cancer tissue tended to be hypomethylated (Additional file 2: Figure S5). With chromosome-wide views, CGI-rich regions were found to be specifically hypermethylated, while repeat-rich regions were widely hypomethylated (Figure 2A; Additional file 2: Figure S6). To analyze methylation changes in genomic elements, we aligned each element at the start and end sites and then obtained the average MES at each respective position. Strikingly, we detected hypermethylation in the upstream region, particularly from 500 bp upstream to the transcription start site (Figure 2B). This is in accordance with the hypermethylation of promoter regions frequently observed in cancer.

The region centered at the transcription start site showed completely different patterns depending on the presence of a CGI, reflecting the low methylation status of CGI-containing promoters (Figure 2C). We also found that, in cancerous tissue, remarkable hypermethylation of CGI-containing promoters occurs and that the density of CpGs is crucial for the increase in DNA methylation (Figure 2C). To further analyze whether 5' regions of genes were hypermethylated similarly to gene promoters, we checked the methylation pattern of the first exons. Interestingly, we found that the first exon was hypermethylated only when it was the 5'-end of a coding exon, but not when it was a 5' UTR exon (Figure 2D). These regions also contained high CpG density. Therefore, CGIs at the upstream regions of genes, the promoter, and the coding start appear to be the major targets of DNA hypermethylation in cancer.

### Methylation pattern of CpG islands

**Figure 3:**
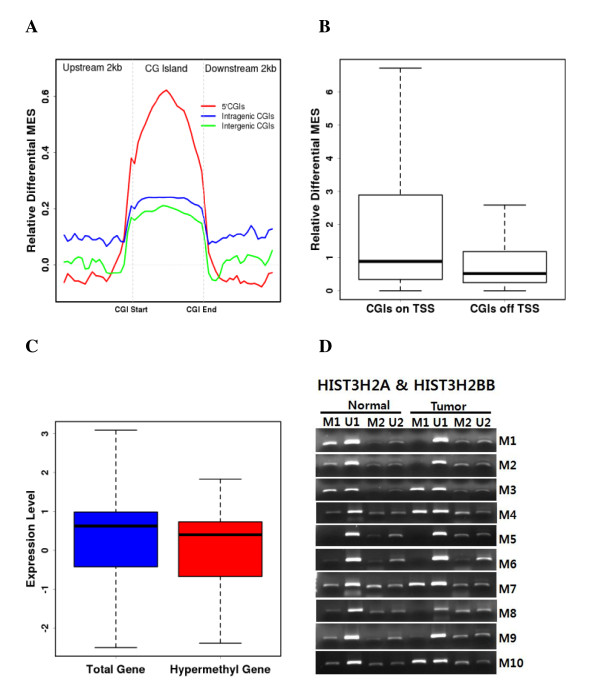
**DNA methylation of CpG islands**. (A) Relative differential MES of subgrouped CGIs. (B) Correlation between differential CGI methylation and distance to the transcription start site. (C) Correlation between gene expression level and the hypermethylation of CGIs. (D) Methylation-specific PCR of histone genes showing the highest differential MES values. M1 and U1 correspond to HIST3H2A, while M2 and U2 correspond to HIST3H2B.

To explore the correlation between the location of CGIs and DNA methylation, we subgrouped CGIs according to their position within the genome. Specifically, they were categorized as 5' (located between 1 kb upstream and the coding start site of a gene), intragenic (intragenic CGIs outside the 5' end), and intergenic (located in non-genic region) (Additional file 1: Table S5). Although CpG density was similar among the three groups, non-5' CGIs (intragenic and intergenic CGIs) were significantly more methylated than 5' CGIs (Additional file 2: Figure S7). We further compared the average MES of subgrouped CGIs and found the methylation of all CGIs was generally increased. However, the relative differential MES suggested that the change in methylation in 5' CGIs was significantly greater than that for other CGIs (Figure 3A), reflecting the important roles of 5' CGIs in cancer. The extent of 5' CGI hypermethylation significantly correlated with the overlap of the transcription start site (Figure 3B).

To explore the functions of genes undergoing differential methylation at 5' CGIs, we selected genes with highly differential CGI MESs (differential MES > 1). We then performed gene ontology (GO) analysis to gain insight into the mechanisms responsible in cancer (Table 3). When the genes were clustered into various GO categories, we found that *HOX *gene clusters and nucleosome assembly-related gene clusters were targets for hypermethylation, while apoptosis-related gene clusters were targets for hypomethylation. Interestingly, our finding that *HOX *gene clusters were preferential targets for DNA methylation is consistent with a previous report [40]. In addition, gene plots confirmed that hypermethylation was CGI-specific in cancer (Additional file 2: Figure S8). To estimate the changes in expression patterns caused by hypermethylation of 5' CGIs, we performed a functional analysis of gene expression data obtained from cDNA microarray experiments. Hypermethylation of 5' CGIs was significantly correlated with downregulation of genes (*p *= 0.03) (Figure 3C; Additional file 3: Table S6 and S7). This indicates that silencing of genes by methylation can be directly affected by the degree of CpG density and 5' CGI hypermethylation. We analyzed the DNA methylation status of genes with hypermethylated 5' CGIs and downregulated expression patterns. Among these was the gene encoding histone H2B type 3-B (*HIST3H2BB*). Analysis of *HIST3H2BB *promoter methylation using methylation-specific PCR revealed that most cancer patients (8/10, 80%) exhibited increased methylation in the promoter region (Figure 3D).

**Table 3:** Functional annotation clustering of genes with hypermethylated 5'CGIs

Annotation Cluster 1	Enrichment Score: 3.27	Count	P_Value
GOTERM_BP_FAT	nucleosome assembly	11	3.90E-04

GOTERM_BP_FAT	chromatin assembly	11	5.20E-04

GOTERM_BP_FAT	protein-DNA complex assembly	11	7.40E-04

**Annotation Cluster 2**	**Enrichment Score: 2.92**	**Count**	**P_Value**

INTERPRO	Histone core	8	6.80E-04

SP_PIR_KEYWORDS	nucleosome core	8	8.60E-04

GOTERM_CC_FAT	nucleosome	8	3.10E-03

**Annotation Cluster 3**	**Enrichment Score: 2.16**	**Count**	**P_Value**

INTERPRO	Homeobox, conserved site	17	5.10E-03

INTERPRO	Homeobox	17	5.70E-03

SP_PIR_KEYWORDS	Homeobox	17	5.80E-03

INTERPRO	Homeodomain-related	17	6.40E-03

SMART	HOX	17	1.40E-02

**Annotation Cluster 4**	**Enrichment Score: 2.02**	**Count**	**P_Value**

PIR_SUPERFAMILY	brain-expressed X-linked protein	3	9.10E-03

PIR_SUPERFAMILY	PIRSF008633:BEX	3	9.10E-03

INTERPRO	Brain expressed X-linked like protein	3	1.00E-02

**Annotation Cluster 5**	**Enrichment Score: 1.97**	**Count**	**P_Value**

PIR_SUPERFAMILY	PIRSF002051:histone H3	3	9.10E-03

INTERPRO	Histone H3	3	1.00E-02

SMART	H3	3	1.30E-02

**Annotation Cluster 6**	**Enrichment Score: 1.78**	**Count**	**P_Value**

UP_SEQ_FEATURE	domain:Helix-loop-helix motif	10	1.20E-02

INTERPRO	Basic helix-loop-helix dimerisation region bHLH	10	1.40E-02

SMART	HLH	10	2.60E-02

**Annotation Cluster 7**	**Enrichment Score: 1.77**	**Count**	**P_Value**

GOTERM_CC_FAT	focal adhesion	9	1.30E-02

GOTERM_CC_FAT	cell-substrate adherens junction	9	1.60E-02

GOTERM_CC_FAT	cell-substrate junction	9	2.20E-02

**Annotation Cluster 8**	**Enrichment Score: 1.55**	**Count**	**P_Value**

GOTERM_BP_FAT	regulation of B cell proliferation	5	1.20E-02

GOTERM_BP_FAT	positive regulation of B cell activation	5	2.20E-02

GOTERM_BP_FAT	regulation of B cell activation	5	8.40E-02

**Annotation Cluster 9**	**Enrichment Score: 1.55**	**Count**	**P_Value**

PIR_SUPERFAMILY	PIRSF500606:homeotic protein Hox D4	3	9.10E-03

SP_PIR_KEYWORDS	embryo	3	3.30E-02

PIR_SUPERFAMILY	PIRSF002612:homeotic protein Hox A5/D4	3	3.70E-02

INTERPRO	Homeobox protein, antennapedia type	3	5.90E-02

**Annotation Cluster 10**	**Enrichment Score: 1.38**	**Count**	**P_Value**

GOTERM_MF_FAT	substrate specific channel activity	21	3.40E-02

GOTERM_MF_FAT	channel activity	21	4.60E-02

GOTERM_MF_FAT	passive transmembrane transporter activity	21	4.70E-02

### DNA methylation of repetitive elements

**Figure 4:**
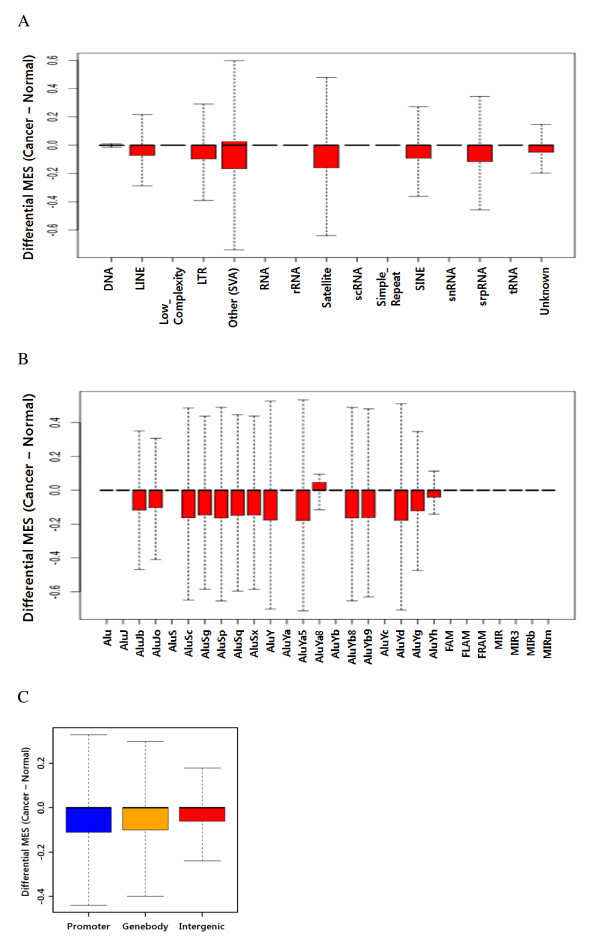
**DNA methylation of repetitive elements**. (A) Differential MES (expressed as Cancer MES - Normal MES) for repeats of various classes. (B) Differential MES for Alu subfamilies. (C) Differential MES for repetitive elements grouped according to their position in the genome.

DNA hypomethylation of repetitive elements is a common feature of cancer. The methylation status of individual repetitive elements such as Alu, LINE-1, and Sat2 is a major target of global DNA methylation studies. However, a comprehensive view of the methylation status of all repetitive elements is currently unavailable. We first checked the methylation status of various repetitive elements to verify the changes in each repetitive element in cancer. When we compared the DNA methylation levels in normal and cancerous tissue, striking changes were observed in the distribution of repeat methylation in the cancerous tissue (Figure 4A).

In addition to the hypomethylation patterns of the SINE and LINE, a significant reduction in methylation was found in SVA (SINE-VNTR-Alus), satellites, and LTR. This is consistent with previous reports about cancer-specific hypomethylation [41]. Alu elements are the most abundant class of repetitive elements in the human genome, with these elements having over one million copies and spanning over 30 lineages. AluS and AluY elements, which are younger subfamilies, were significantly hypomethylated when compared with older subfamilies, as previously reported [42] (Figure 4B). SVA elements, which have been extensively mobilized in the human genome, consist of a combination of sequences derived from other retroelements [43].

To understand if a correlation may exist between the degree of methylation changes and genomic location, we analyzed the methylation of repetitive elements based on their genomic location (*i.e.*, promoter, gene body, or intergenic), even though it is unclear if repetitive elements participate in regulating gene expression (Figure 4C). We found that methylation changes were higher in gene-associated repetitive elements than in gene-independent repetitive elements. This suggests that a correlation exists between methylation changes in repetitive elements and the expression of adjacent genes.

### Long-range epigenetic silencing (LRES) in gastric cancer

**Figure 5:**
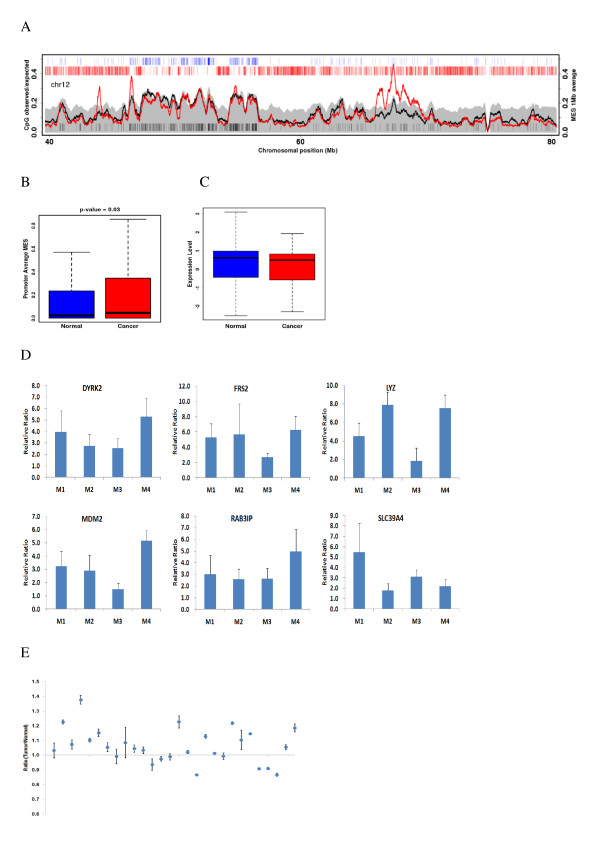
**DNA methylation of LRES regions**. (A) Average MES curve for LRES regions in chromosome 12 in normal (black) and cancerous (red) tissue. (B) Average MESs for gene promoters in LRES regions in normal and cancerous tissue. (C) Correlation between gene expression levels and hypermethylation of genes within LRES regions. (D) Methylation enrichment of several selected genes within LRES regions, as assessed by MIRA-qPCR. (E) Methylation ratio at the target site within the upstream region of *MDM2*, as measured by pyrosequencing.

Large chromosomal regions can usually be suppressed in cancer cells, as seen by hypermethylation of neighboring CpG islands and downregulation of most genes within the region. To determine whether LRES occurs in gastric cancer, we identified large-scale genomic regions where methylation enrichment encompasses multiple genes. Interestingly, we found a high degree of hypermethylation around the 12q14 site, even though there were few CGIs and genes with abundant repeats (Figure 5A).

The LRES region around the 12q14 site spanned about 2.7 Mb and harbored about 21 genes. Among these genes was *MDM2*, which encodes a protein that is considered to be a negative regulator of *p53 *and a major regulator of cancer development. Promoters of genes in this LRES region displayed tumor-specific hypermethylation (*p *= 0.03, t-test) (Figure 5B). To determine whether genes in this LRES region show concordant gene silencing, we re-analyzed publicly available expression datasets (GSE27342) for differential gene expression in clinical samples. Consistent with previously reported expression patterns of LRES regions, the public data for this LRES region showed common gene suppression (*p *= 0.05, t-test) (Figure 5C).

To determine if the hypermethylation pattern of genes within this LRES region is commonly present in gastric cancer, we examined the methylation enrichment frequency of selected genes (Figure 5D). The methylation enrichment levels of cancer samples were over 2-fold higher than that of normal samples. However, one of the patients showed a low methylation level at several target sites (Additional file 2: Figure S9). Because the amplification of this region frequently occurs in many cancers, we examined the amplification frequency of several genes within these regions using real-time PCR. Intriguingly, we detected amplification of *MDM2 *(Additional file 2: Figure S10), suggesting that an interaction exists between DNA methylation and gene amplification. To examine the generality of *MDM2 *methylation, we used pyrosequencing to analyze the methylation level of a specific locus in an upstream region of *MDM2*. We analyzed *MDM2 *methylation in normal and cancer tissue samples from other 28 gastric cancer patients. Out of 28 independent samples, most patients showed higher *MDM2 *methylation (Figure 5E), while four samples showed decreased levels of methylation, along with gene amplification (Additional file 2: Figure S11). Therefore, the *MDM2*-containing region appears to be hypermethylated in cancer in general, but our results suggest that gene amplification in this region interferes with methylation. Therefore, except for cases with gene amplification, LRES across 12q14 appears to be a distinct epigenetic pattern associated with gastric cancer.

### Methylation patterns in metastatic lymph nodes

**Figure 6:**
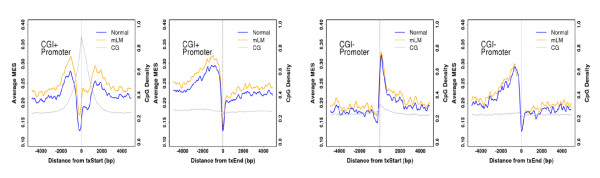
**Comparison of methylation patterns in normal and metastatic lymph nodes**. DNA methylation at transcript ends and coding region ends is shown.

In gastric cancer, lymph node metastasis is one of the major prognostic factors and an important indicator of tumor aggressiveness. Many studies have been conducted to analyze the expression profiles for and epigenetic changes in specific genes, but comprehensive data for whole-genome alterations in DNA methylation remain scarce. Similar to cancerous tissue, metastatic lymph nodes showed hypermethylation at the region centered at the transcription start site only in CGI-containing promoters (Figure 6). GO analysis revealed that several clustered groups of genes with highly differential CGI MES (differential MES > 1) in 5' CGIs overlapped with cancer genes, such as HOX family and histone family genes, even if there was little difference in ranks (Additional file 1: Table S8). After considering all these data, we conclude that the methylation pattern of metastatic lymph nodes is considerably similar to that of the cancer of origin.

## Discussion

Here we demonstrate a comprehensive methylation map of human gastric tissue at high resolution. Our data provide a global view of a mammalian methylome, along with several intriguing findings, some of which are novel and worth further investigation. First, we found that hypermethylation of CGIs in promoters is an important epigenetic feature that dictates gene expression changes in cancer. Second, hypermethylation of the 5'-end of coding exons arises in cancer and appears to play an important role in cancer progression. Third, cancer-induced methylation changes in younger repetitive elements and LRES have potential clinical implications in terms of early detection and therapeutic design.

Among the genes analyzed in this study was *MDM2*, which encodes an important negative regulator of p53. *MDM2 *and *p53 *are known to regulate one another through a feedback loop [44]. *MDM2 *overexpression is frequently detected in many human cancers, suggesting that *MDM2 *overexpression may be one of the common features of tumorigenesis. In this study, we showed that the upstream region of *MDM2 *is hypermethylated in most cancer samples. We also found that, in some cancer samples, hypomethylation occurred along with *MDM2 *amplification at the same site, suggesting that there is major dysregulation of the *MDM2*-mediated pathway at both the genetic and epigenetic levels. This appears to cause aberrant early tumor cell development and subsequently cancer.

*HOX *genes cluster on chromosomes 2, 7, 12, and 17, and they are frequently inactivated by CpG hypermethylation in several cancers [40,45,46]. Accordingly, we found that many *HOX *genes were hypermethylated, indicating that *HOX *gene clusters may be general targets of epigenetic alterations during tumorigenesis. However, hypomethylation of a few *HOX *genes was also detected in gastric cancer. Therefore, the methylation of *HOX *genes may be regulated in a tissue-specific manner in cancer. In addition, the regulation of *HOX *expression is significantly correlated with histone modifications and interaction with Polycomb group genes [47,48]. Our data point to another possible mode of regulating *HOX *gene expression-- DNA hypermethylation of the promoter region of histone genes such as H2B. Further investigation of histone genes might offer new insights for cancer studies.

DNA hypomethylation of repetitive elements as a major contributor to genome size is one of common features in cancer and the methylation changes in SINE, LINE, or LTR-retrotransposon with possess transcriptional activity are critical for cellular functions. A number of SINEs close to CpG islands retain a high proportion of CpG sites and frequently hypomethylated in cancer. Because younger Alu elements are usually closer to active chromatin regions [49], the hypomethylation of them has more biological significance than that of older Alu elements. Additionally, these hypomethylation of repeat elements, such as Alu and LINE1 might also affect the inactivation of X chromosome [50].

The findings in this study are based on an intuitive and efficient normalization method for comparative analysis. Unlike bisulfite sequencing, MIRA and MeDIP simulate the *in vivo *behavior of methyl-CpG binding domain (MBD) proteins, which recognize both the methylation level and concentration of individual CpG sites [38]. For example, it has been shown that MBD binding is not sufficient for gene repression at low CpG densities, even when individual sites are highly methylated. In this case, the common practice is to use MeDIP or MIRA outputs as measures for the functional consequences of methylation of all CpGs in a given region [26,30,32,38,51-53]. However, a few studies have attempted to use the spatial density of CpGs to normalize the experimental readout of MeDIP-chip [25,54]. Therefore, the MES normalization used here provides several advantages over other methods. First, using logarithmic transformation, we can scale down raw read counts four orders of magnitude to obtain MESs. This provides mathematical benefits such as variance stabilization. Second, MES normalization allows us to use the correct background distribution. As a probability function for the number of events in a given time interval, a Poisson distribution can be used to assess the statistical significance of read counts in a given genomic interval. However, we found that our MES index, particularly when normalized with local methods rather than raw read counts, better illustrates the nature of the Poisson event. Third and most importantly, it enables comparative analysis of independent samples. In the normalization step, direct subtraction of MESbg proves to be an efficient correction method for background sequencing.

Although the methods used here have clear advantages, the biological and technical limitations of these methods should also be mentioned. Since our methods are based on affinity purification, methylation changes and karyotypic alterations cannot be distinguished. However, this can be overcome by comparing normal and cancer genomes following measurement of background enrichment. Thus, this comparative analysis scheme should be of value for future clinical epigenomics investigations.

## Conclusions

We have generated high resolution genome-wide map of human gastric cancer by MIRA-seq, and have found that 5' CGIs and the 5'-end of coding exons are hypermethylated. Hypermethylation of 5' CGIs was significantly correlated with downregulation of associated genes. We found novel long-range epigenetic silencing (LRES) regions and identified several hypermethylated genes (MDM2, DYRK2, and LYZ) within these regions. The methylation status of metastatic lymph nodes was intermediate between normal and cancerous tissue, indicating that methylation is gradually increased in tumorigenesis. Our method is readily applicable to a comparative analysis of methylomes and other types of epigenomic data.

## Competing interests

The authors declare that they have no competing interests.

## Authors' contributions

JHP conceived of the study, carried out the analysis, and wrote the manuscript. JAP performed MSP and pyrosequencing. JKC participated in sequencing data analysis. JL, MGB, YGL processed the raw sequencing data and participated in data analysis. JBB carried out the purification and sequencing of methylated DNA. DYP participated in microarray data analysis. HKY participated in sample preparation. TYK and YJK participated in study design and coordination, and finalized the manuscript. All authors read and approved the final manuscript.

## Pre-publication history

The pre-publication history for this paper can be accessed here:

http://www.biomedcentral.com/1755-8794/4/82/prepub

## Supplementary Material

Additional file 1 **Supplementary Tables 1**. Supplementary Table 1: Primers for pyrosequencing, MSP and MIRA-qPCR. Supplementary Table 2: Number of uniquely matched reads (U0, U1, and U2 from ELAND alignment results) in each experiment. Supplementary Table 3: Number of covered genomic CpG sites and total CpG counts in our methylome. Supplementary Table 5: Comparison of 5', intragenic and intergenic CGIs. Supplementary Table 8: Functional annotation clustering of genes with hypermethylated 5'CGIs in metastatic lymph nodes. Click here for file

Additional file 2 **Supplementary Figures**. Supplementary Figure 1: Sensitivity of the MIRA technique. Supplementary Figure 2: The distribution of MESs from MIRA-seq, input and randomized methylome of normal. Supplementary Figure 3: Goodness of fit was tested for two basic normalized MESs (MESt and MESl), two background normalized MESs (MESt-MESbg and MESl-MESbg), and raw readcounts against the Poisson and Gaussian model. Supplementary Figure 4: Average MES pattern of human promoters which were subgrouped into high, intermediate, and low CpG density promoters (HCP, ICP, and LCP, respectively). Supplementary Figure 5: Chromosome-wide average MESs for normal (black circle) and cancerous (red circle) tissue. Supplementary Figure 6: Chromosomal distribution of the average MES (black (normal) and red (cancer) curve corresponding to the left axis) and the average CpG observed/expected ratio (gray shade corresponding to the left axis) in 1 Mb sliding windows. Supplementary Figure 7: Average MES pattern of subgrouped CGIs (5'CGIs, intergenic CGIs and intergenic CGIs). Supplementary Figure 8: Plots of several genes with 5'CGIs hypermethylation. Supplementary Figure 9: *MDM2 *methylation level of normal and cancerous tissue in three individual samples by pyrosequencing. Supplementary Figure 10: Amplification ratio of several genes (*DYRK2*, *IFNG*, *IL26*, *MDM1*, and *MDM2*) within chromosome 12q14 LRES region by real-time qPCR. Supplementary Figure 11: Amplification ratio of specific locus in an upstream region of *MDM2 *by real-time qPCR. Click here for file

Additional file 3 **Supplementary Tables 2**. Supplementary Table 4, 6, 7. Supplementary Table 4: Promoter class according to CpG density. Supplementary Table 6: The genes with hypermethylation patterns and low expression levels. Supplementary Table 7: The genes with hypomethylation patterns and high expression levels. Click here for file
