# Supplementary material for: Identification of DNA methylation changes associated with human gastric cancer
Source: BMC Med Genomics. 2011 Dec 2;4:82. doi: 10.1186/1755-8794-4-82 (PMC3273443; doi:10.1186/1755-8794-4-82)
Supplement: Additional file 1 — Supplementary Tables 1. Supplementary Table 1: Primers for pyrosequencing, MSP and MIRA-qPCR. Supplementary Table 2: Number of uniquely matched reads (U0, U1, and U2 from ELAND alignment results) in each experiment. Supplementary Table 3: Number of covered genomic CpG sites and total CpG counts in our methylome. Supplementary Table 5: Comparison of 5', intragenic and intergenic CGIs. Supplementary Table 8: Functional annotation clustering of genes with hypermethylated 5'CGIs in metastatic lymph nodes. [file 1755-8794-4-82-S1.PDF]

Supplementary Table 1

Primers for pyrosequencing, MSP and MIRA-qPCR

Pyrosequencing

MDM2 forward	TTTATTATTTTGGTTAGGTTGGTTT
MDM2 reverse	Biotin-CATCAAATCATATACAATAAAATTTCTAAT
MDM2 sequencing	GTTTTTTAAAGTGTTGGGATTA

Methylation-specific PCR

HIST3H2BA-F (unmethyl)	GATGTTGGGTAGGATGTTATTTTGT
HIST3H2BA-R (unmethyl)	CCCAATCTATCTAACCACAATACTCA
HIST3H2BA-F (methyl)	GTTGGGTAGGACGTTATTTTGC
HIST3H2BA-R (methyl)	GATCTATCTAACCGCGATACTCG
HIST3H2BB-F (unmethyl)	AAGTAGGTGTATTTTGATATTGGTATTTTG
HIST3H2BB-R (unmethyl)	ACCATCTACACTTCACAAAACATA
HIST3H2BB-F (methyl)	AGGTGTATTTCGATATCGGTATTTC
HIST3H2BB-R (methyl)	GTCTACACTTCGCGAAACGTA

MIRA-qPCR

MDM2-F (Target)	CAGCCTCCCAAGTGGCTA
MDM2-R (Target)	GGTGGATCACGAGGTTAGGA
MDM2-F (Control)	CTGGCCAGAGAAGAAACGTG

MDM2-R (Control)	AACGTTTTTGATGCGGTCTC
SLC39A4-F (Target)	TGCAGGAATACCTGGGAGAG
SLC39A4-R (Target)	CAAGGCCCAGTGCTTCTG
SLC39A4-F (Control)	TGCCCCAATCTTATCCTGAC
SLC39A4-R (Control)	AGGAAGCTCCAACACAGACAA
LYZ-F (Target)	GGTTATTTGTGCCCATCCAA
LYZ-R (Target)	ACCAGGACAGCACCAAGC
LYZ-F (Control)	GTTCAGGTTGGCAAGCAAAT
LYZ-R (Control)	TGCTGGTTTCAGCCTTATGC
DYRK2-F (Target)	CACCGGAGAGCAGAGAGTG
DYRK2-R (Target)	TGCAGGATCCCACTCTAAACA
DYRK2-F (Control)	CATTCTAAAAGCCGGCAAAG
DYRK2-R (Control)	ACAAAACAGCTCACCGATCA
RAB3IP-F (Target)	GGACGGCTGGGCAGA
RAB3IP-R (Target)	GCCCAGCCACCCTGTC
RAB3IP-F (Control)	TTTGCTCATTAGTTGATGCAGTT
RAB3IP-R (Control)	CAACCGGTACCAGCCACT
FRS2-F (Target)	TGAGATTGAGGCTGCAGTGA
FRS2-R (Target)	TTTTTCCCCTTTGAGACAGG
FRS2-F (Control)	AAATTAGCCAGGGATGTTGG
FRS2-R (Control)	CTGCAGCCTCCACCTCCT

Supplementary Table 2

Number of uniquely matched reads (U0, U1, and U2 from ELAND alignment results) in each experiment

Sample	Replicate	# Reads
	Experiment 1	6,822,179
Normal	Experiment 2	2,964,286
	**Total**	**9,786,465**
	Experiment 1	5,527,253
Cancer	Experiment 2	5,686,554
	**Total**	**11,213,807**
Metastatic Lymph Node	Experiment 1	**5,657,927**
Normal Input	Experiment 1	**7,990,327**

Supplementary Table 3

Number of covered genomic CpG sites and total CpG counts in our methylome. The total number of CpG dinucleotides in the human genome (except centromeres) is 28,163,863. Up to 48% of them were detected by our MIRA-seq experiments at least one time (the first row). Many of them were read more than one time, leading to ~ 7.01x coverage for each CpG site on average (the second row).

**Total**	**Normal**	**Cancer**	**Metastasis Lymph Node**
Number of genomic CpG sites covered by our MIRA-seq (percentage to the total number of CpGs in the genome)	**13,619,933 (48.4 %)**	**11,808,464 (41.9%)**	**11,000,422 (39.1%)**
Total CpG counts in our methylome (average ratio of each covered CpG site)	**67,737,282 (4.97 X)**	**82,871,337 (7.01 X)**	**49,920,386 (4.53 X)**

Supplementary Table 5

Comparison of 5’, intragenic and intergenic CGIs. The mean values and the P value from the two-sample t-test are shown.

Feature	5’CGIs	Intragenic CGIs	P-value
Length	949.8	532.4	3.02E-233
CpG Number	88.3	46.3	0
GC Number	614.0	352.2	4.86E-255
CpG Percentage	19.1	17.5	1.12E-247

Feature	5’CGIs	Intergenic CGIs	P-value
Length	949.8	604.4	2.83E-80
CpG Number	88.3	54.0	2.12E-141
GC Number	614.0	396.1	3.33E-91
CpG Percentage	19.1	18.2	4.72E-67

Supplementary Table 8

Functional annotation clustering of genes with hypermethylated 5′CGIs in metastatic lymph nodes

**Annotation Cluster 1**	**Enrichment Score: 6.06**	**Count**	**P_Value**	**Benjamini**
GOTERM_BP_FAT	regulation of nucleotide biosynthetic process	21	6.20E-07	3.20E-04
GOTERM_BP_FAT	regulation of cyclic nucleotide biosynthetic process	21	6.20E-07	3.20E-04
GOTERM_BP_FAT	regulation of cyclic nucleotide metabolic process	21	9.70E-07	3.10E-04
GOTERM_BP_FAT	regulation of nucleotide metabolic process	21	1.50E-06	3.50E-04
**Annotation Cluster 2**	**Enrichment Score: 5.58**	**Count**	**P_Value**	**Benjamini**
GOTERM_BP_FAT	regulation of adenylate cyclase activity	19	1.40E-06	3.70E-04
GOTERM_BP_FAT	regulation of cyclase activity	19	2.30E-06	4.90E-04
GOTERM_BP_FAT	regulation of lyase activity	19	3.00E-06	5.20E-04
GOTERM_BP_FAT	regulation of cAMP biosynthetic process	19	3.00E-06	5.20E-04
GOTERM_BP_FAT	regulation of cAMP metabolic process	19	4.10E-06	5.50E-04
**Annotation Cluster 3**	**Enrichment Score: 5.55**	**Count**	**P_Value**	**Benjamini**

GOTERM_BP_FAT	negative regulation of cyclase activity	14	2.80E-06	5.20E-04
GOTERM_BP_FAT	negative regulation of lyase activity	14	2.80E-06	5.20E-04
GOTERM_BP_FAT	negative regulation of adenylate cyclase activity	14	2.80E-06	5.20E-04
**Annotation Cluster 4**	**Enrichment Score: 4.22**	**Count**	**P_Value**	**Benjamini**
INTERPRO	Homeobox, conserved site	28	2.80E-05	5.50E-03
SP_PIR_KEYWORDS	Homeobox	28	3.00E-05	1.50E-03
INTERPRO	Homeobox	28	3.50E-05	5.90E-03
SMART	HOX	28	4.40E-04	3.30E-02
**Annotation Cluster 5**	**Enrichment Score: 2.98**	**Count**	**P_Value**	**Benjamini**
INTERPRO	Peptidase M12B, ADAM-TS	8	1.10E-04	1.40E-02
UP_SEQ_FEATURE	domain:TSP type-1 4	8	5.00E-04	4.60E-02
UP_SEQ_FEATURE	domain:TSP type-1 3	8	1.30E-03	9.10E-02
UP_SEQ_FEATURE	domain:TSP type-1 2	8	4.30E-03	2.50E-01
UP_SEQ_FEATURE	domain:TSP type-1 1	8	4.30E-03	2.50E-01
**Annotation Cluster 6**	**Enrichment Score: 2.84**	**Count**	**P_Value**	**Benjamini**
INTERPRO	Potassium channel, voltage dependent, Kv	8	2.50E-04	2.60E-02

INTERPRO	Voltage-dependent potassium channel	8	9.30E-04	7.50E-02
INTERPRO	Potassium channel, voltage dependent, Kv, tetramerisation	8	1.30E-02	4.90E-01
**Annotation Cluster 7**	**Enrichment Score: 2.43**	**Count**	**P_Value**	**Benjamini**
GOTERM_BP_FAT	activation of adenylate cyclase activity by G-protein signaling pathway	8	3.70E-03	1.90E-01
GOTERM_BP_FAT	positive regulation of adenylate cyclase activity by G-protein signaling pathway	8	3.70E-03	1.90E-01
GOTERM_BP_FAT	regulation of adenylate cyclase activity involved in G-protein signaling	8	3.70E-03	1.90E-01
**Annotation Cluster 8**	**Enrichment Score: 2.24**	**Count**	**P_Value**	**Benjamini**
INTERPRO	Diacylglycerol kinase accessory region	5	9.50E-04	7.20E-02
SMART	DAGKa	5	1.70E-03	9.60E-02
UP_SEQ_FEATURE	domain:DAGKc	5	4.50E-03	2.50E-01
INTERPRO	Diacylglycerol kinase, catalytic region	5	5.10E-03	3.10E-01
GOTERM_MF_FAT	diacylglycerol kinase activity	5	5.80E-03	1.70E-01
SMART	DAGKc	5	8.90E-03	2.90E-01
**Annotation Cluster 9**	**Enrichment Score: 2.11**	**Count**	**P_Value**	**Benjamini**
GOTERM_BP_FAT	activation of adenylate cyclase activity	9	6.40E-03	2.40E-01

GOTERM_BP_FAT	positive regulation of adenylate cyclase activity	9	7.10E-03	2.60E-01
GOTERM_BP_FAT	positive regulation of cyclase activity	9	8.00E-03	2.70E-01
GOTERM_BP_FAT	positive regulation of lyase activity	9	9.80E-03	2.90E-01
**Annotation Cluster 10**	**Enrichment Score: 2.1**	**Count**	**P_Value**	**Benjamini**
UP_SEQ_FEATURE	domain:SCAN box	9	5.10E-03	2.80E-01
INTERPRO	Transcriptional regulator SCAN	9	6.30E-03	3.40E-01
SMART	SCAN	9	1.60E-02	3.70E-01
**Annotation Cluster 11**	**Enrichment Score: 2.07**	**Count**	**P_Value**	**Benjamini**
UP_SEQ_FEATURE	DNA-binding region:T-box	5	5.70E-03	3.00E-01
INTERPRO	Transcription factor, T-box, conserved site	5	8.20E-03	3.70E-01
INTERPRO	Transcription factor, T-box	5	8.20E-03	3.70E-01
SMART	TBOX	5	1.40E-02	3.80E-01
**Annotation Cluster 12**	**Enrichment Score: 2.02**	**Count**	**P_Value**	**Benjamini**
GOTERM_BP_FAT	neuron projection morphogenesis	22	2.90E-03	1.50E-01
GOTERM_BP_FAT	cell projection morphogenesis	22	1.40E-02	3.60E-01
GOTERM_BP_FAT	cell part morphogenesis	22	2.20E-02	4.40E-01

Annotation Cluster 13	Enrichment Score: 1.99	Count	P_Value	Benjamini
GOTERM_BP_FAT	positive regulation of phospholipase C activity	10	5.50E-03	2.20E-01
GOTERM_BP_FAT	activation of phospholipase C activity	10	5.50E-03	2.20E-01
GOTERM_BP_FAT	positive regulation of phospholipase activity	10	8.10E-03	2.70E-01
GOTERM_BP_FAT	regulation of phospholipase activity	10	9.70E-03	3.00E-01
GOTERM_BP_FAT	positive regulation of lipase activity	10	1.40E-02	3.60E-01
GOTERM_BP_FAT	regulation of lipase activity	10	3.30E-02	5.20E-01
**Annotation Cluster 14**	**Enrichment Score: 1.93**	**Count**	**P_Value**	**Benjamini**
UP_SEQ_FEATURE	domain:Peptidase M12B	7	1.10E-02	4.70E-01
INTERPRO	Peptidase M12B, propeptide	7	1.10E-02	4.50E-01
INTERPRO	Peptidase M12B, ADAM/reprolysin	7	1.10E-02	4.50E-01
UP_SEQ_FEATURE	domain:Disintegrin	7	1.20E-02	5.00E-01
INTERPRO	Disintegrin, conserved site	7	1.40E-02	4.70E-01
**Annotation Cluster 15**	**Enrichment Score: 1.83**	**Count**	**P_Value**	**Benjamini**
PIR_SUPERFAMILY	PIRSF002444:gamma-aminobutyric acid transporter	5	6.20E-03	6.40E-01
INTERPRO	Sodium	5	1.50E-02	4.80E-01

GOTERM_MF_FAT	neurotransmitter:sodium symporter activity	5	1.70E-02	3.90E-01
GOTERM_MF_FAT	neurotransmitter transporter activity	5	3.10E-02	5.40E-01
**Annotation Cluster 16**	**Enrichment Score: 1.81**	**Count**	**P_Value**	**Benjamini**
SP_PIR_KEYWORDS	potassium transport	13	8.00E-03	1.20E-01
SP_PIR_KEYWORDS	potassium	13	1.60E-02	2.20E-01
GOTERM_MF_FAT	potassium ion binding	13	3.00E-02	5.40E-01
**Annotation Cluster 17**	**Enrichment Score: 1.71**	**Count**	**P_Value**	**Benjamini**
GOTERM_BP_FAT	cellular calcium ion homeostasis	18	1.20E-02	3.40E-01
GOTERM_BP_FAT	calcium ion homeostasis	18	1.60E-02	3.80E-01
GOTERM_BP_FAT	cellular metal ion homeostasis	18	2.30E-02	4.50E-01
GOTERM_BP_FAT	metal ion homeostasis	18	3.30E-02	5.20E-01
**Annotation Cluster 18**	**Enrichment Score: 1.64**	**Count**	**P_Value**	**Benjamini**
PIR_SUPERFAMILY	PIRSF002051:histone H3	3	1.90E-02	7.90E-01
INTERPRO	Histone H3	3	2.20E-02	5.60E-01
SMART	H3	3	2.90E-02	5.40E-01
**Annotation Cluster 19**	**Enrichment Score: 1.5**	**Count**	**P_Value**	**Benjamini**

GOTERM_CC_FAT	chromatoid body	3	2.50E-02	3.60E-01
GOTERM_CC_FAT	P granule	3	3.40E-02	4.20E-01
GOTERM_CC_FAT	pole plasm	3	3.40E-02	4.20E-01
GOTERM_CC_FAT	germ plasm	3	3.40E-02	4.20E-01
**Annotation Cluster 20**	**Enrichment Score: 1.39**	**Count**	**P_Value**	**Benjamini**
PIR_SUPERFAMILY	PIRSF501018:sphingosine 1-phosphate receptor 1-5	3	1.90E-02	7.90E-01
INTERPRO	Sphingosine 1-phosphate receptor	3	2.20E-02	5.60E-01
PIR_SUPERFAMILY	PIRSF005435:LPAR1/S1PR1-like lysophospholipid receptor	3	4.80E-02	9.00E-01
