# Supplementary material for: Identification of DNA methylation changes associated with human gastric cancer
Source: BMC Med Genomics. 2011 Dec 2;4:82. doi: 10.1186/1755-8794-4-82 (PMC3273443; doi:10.1186/1755-8794-4-82)
Supplement: Additional file 2 — Supplementary Figures. Supplementary Figure 1: Sensitivity of the MIRA technique. Supplementary Figure 2: The distribution of MESs from MIRA-seq, input and randomized methylome of normal. Supplementary Figure 3: Goodness of fit was tested for two basic normalized MESs (MESt and MESl), two background normalized MESs (MESt-MESbg and MESl-MESbg), and raw readcounts against the Poisson and Gaussian model. Supplementary Figure 4: Average MES pattern of human promoters which were subgrouped into high, intermediate, and low CpG density promoters (HCP, ICP, and LCP, respectively). Supplementary Figure 5: Chromosome-wide average MESs for normal (black circle) and cancerous (red circle) tissue. Supplementary Figure 6: Chromosomal distribution of the average MES (black (normal) and red (cancer) curve corresponding to the left axis) and the average CpG observed/expected ratio (gray shade corresponding to the left axis) in 1 Mb sliding windows. Supplementary Figure 7: Average MES pattern of subgrouped CGIs (5'CGIs, intergenic CGIs and intergenic CGIs). Supplementary Figure 8: Plots of several genes with 5'CGIs hypermethylation. Supplementary Figure 9: MDM2 methylation level of normal and cancerous tissue in three individual samples by pyrosequencing. Supplementary Figure 10: Amplification ratio of several genes (DYRK2, IFNG, IL26, MDM1, and MDM2) within chromosome 12q14 LRES region by real-time qPCR. Supplementary Figure 11: Amplification ratio of specific locus in an upstream region of MDM2 by real-time qPCR. [file 1755-8794-4-82-S2.PDF]

Supplementary Fig. 1

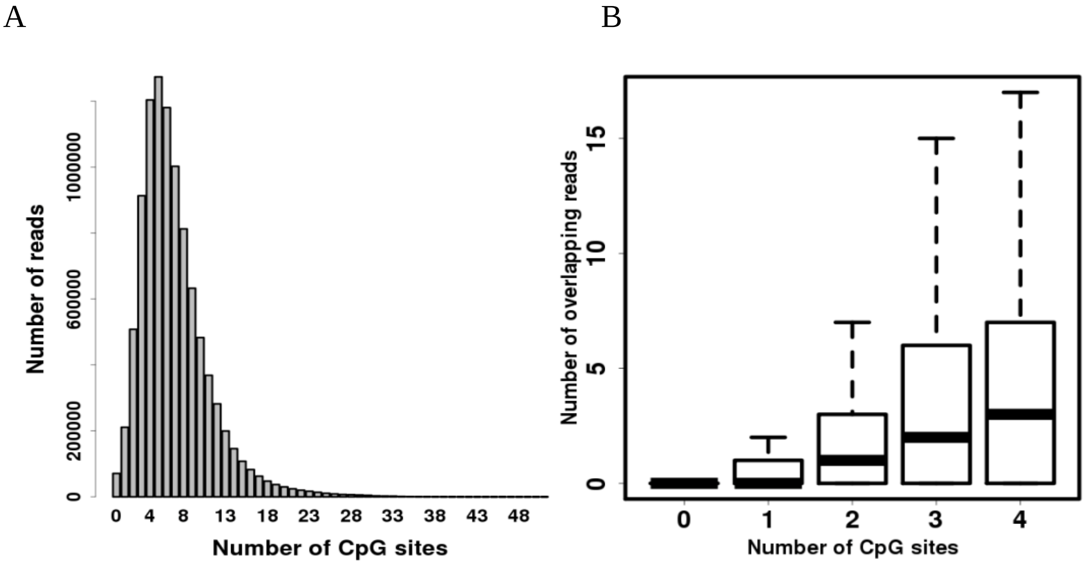

Sensitivity of the MIRA technique. Shown is the number of solexa reads containing CpG sites in their sequence (200bp) (A) and overlapping reads for 50bp genomic intervals containing 0 ~ 4 CpG sites in their sequence (B).

Supplementary Fig. 2

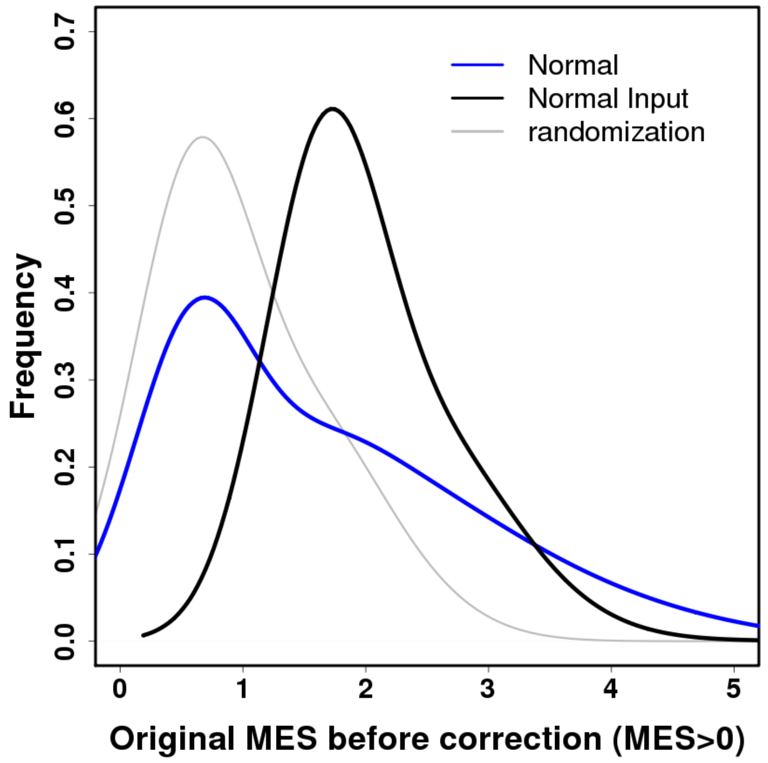

The distribution of MESs from MIRA-seq, input and randomized methylome of normal (permutation of MIRA reads along the chromosome). The original MES values before correction for sequencing bias were used for comparison.

Supplementary Fig. 3

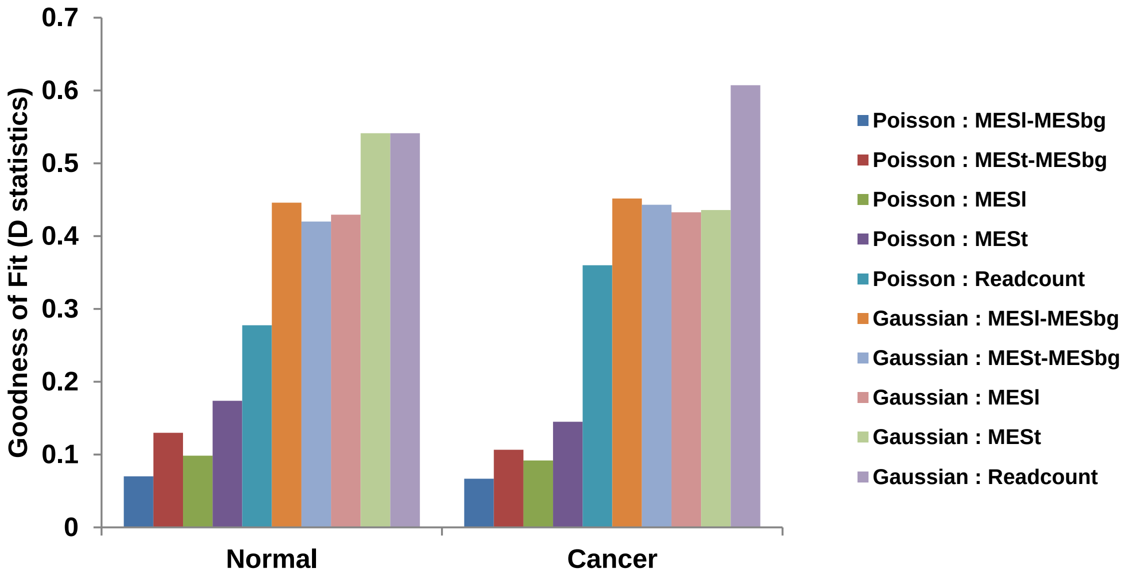

Goodness of fit was tested for two basic normalized MESs (MESt and MESl), two background normalized MESs (MESt-MESbg and MESl-MESbg), and raw readcounts against the Poisson and Gaussian model. The D statistic is from the Kolmogorov-Smirnov test. **MESt**; **MES** normalized by **t**otal readcount, **MESl**; **MES** normalized by **l**ocal readcount, **MESbg**; MES of input sequencing

Supplementary Fig. 4

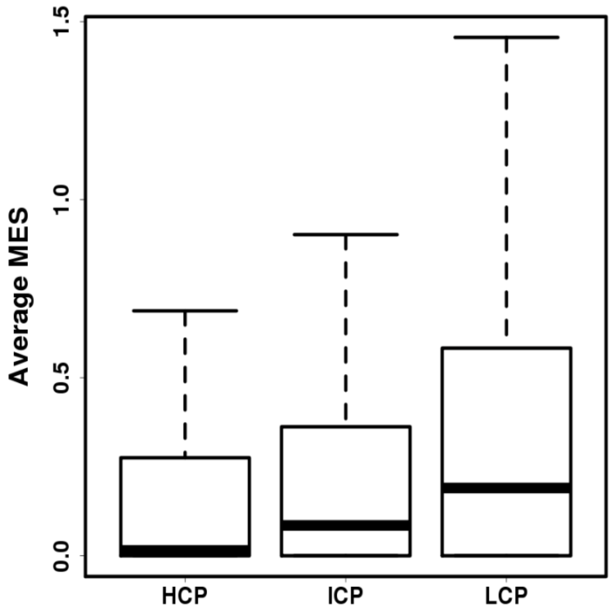

Average MES pattern of human promoters which were subgrouped into high, intermediate, and low CpG density promoters (HCP, ICP, and LCP, respectively).

Supplementary Fig. 5

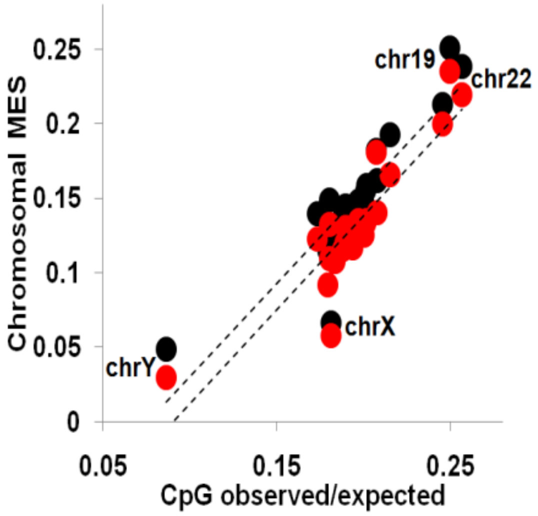

Chromosome-wide average MESs for normal (black circle) and cancerous (red circle) tissue.

Supplementary Fig. 6

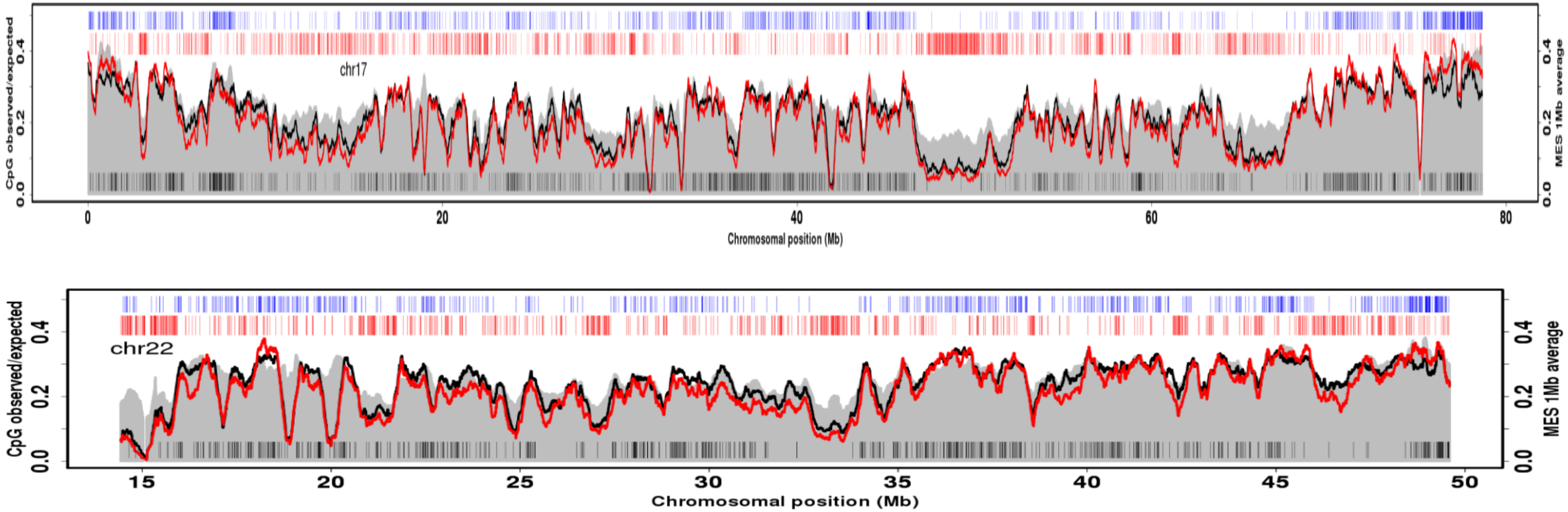

Chromosomal distribution of the average MES (black (normal) and red (cancer) curve corresponding to the left axis) and the average CpG observed/expected ratio (gray shade corresponding to the left axis) in 1 Mb sliding windows. The positions of transcribed genes (black bars at the bottom) and those of long repeats (> 1kb; red bars on the top), CGIs (blue bars on the top) are compared in the context of DNA methylation and CpG density.

Supplementary Fig. 7

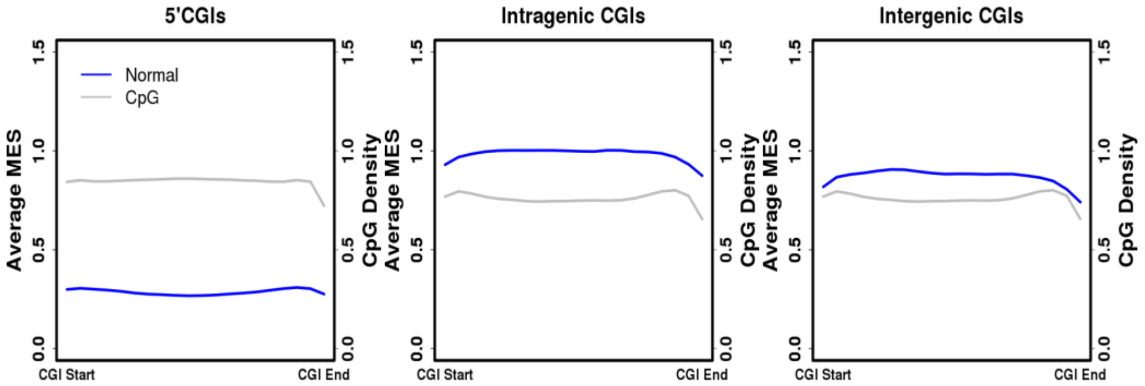

Average MES pattern of subgrouped CGIs (5′CGIs, intergenic CGIs and intergenic CGIs). Each CGI was partitioned into 20 bins, and the average MES was obtained for each bin of all corresponding elements. Blue line; average MES of 5′CGIs in normal sample, Gray line; average CpG density in 5′CGIs region.

Supplementary Fig. 8

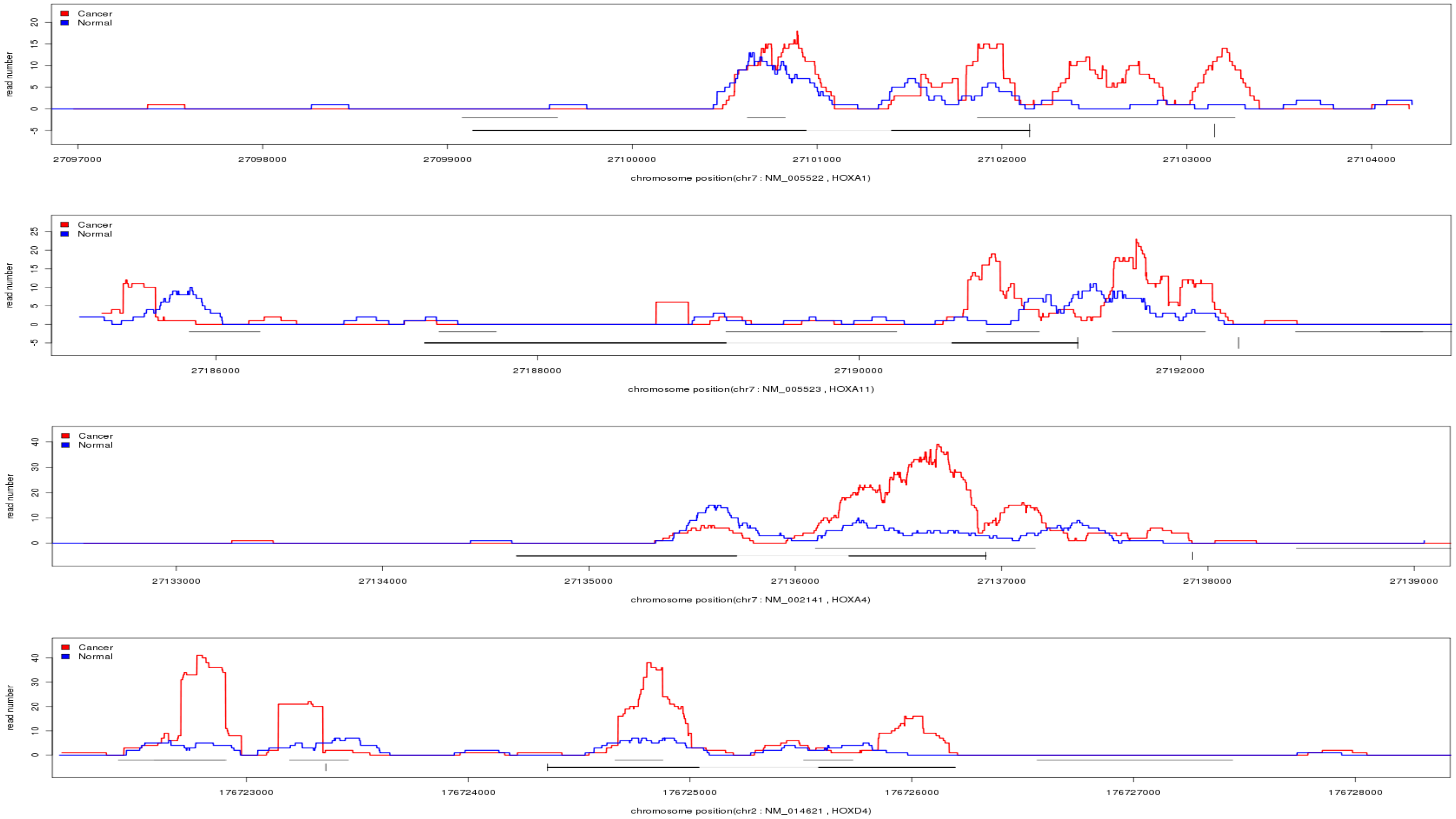

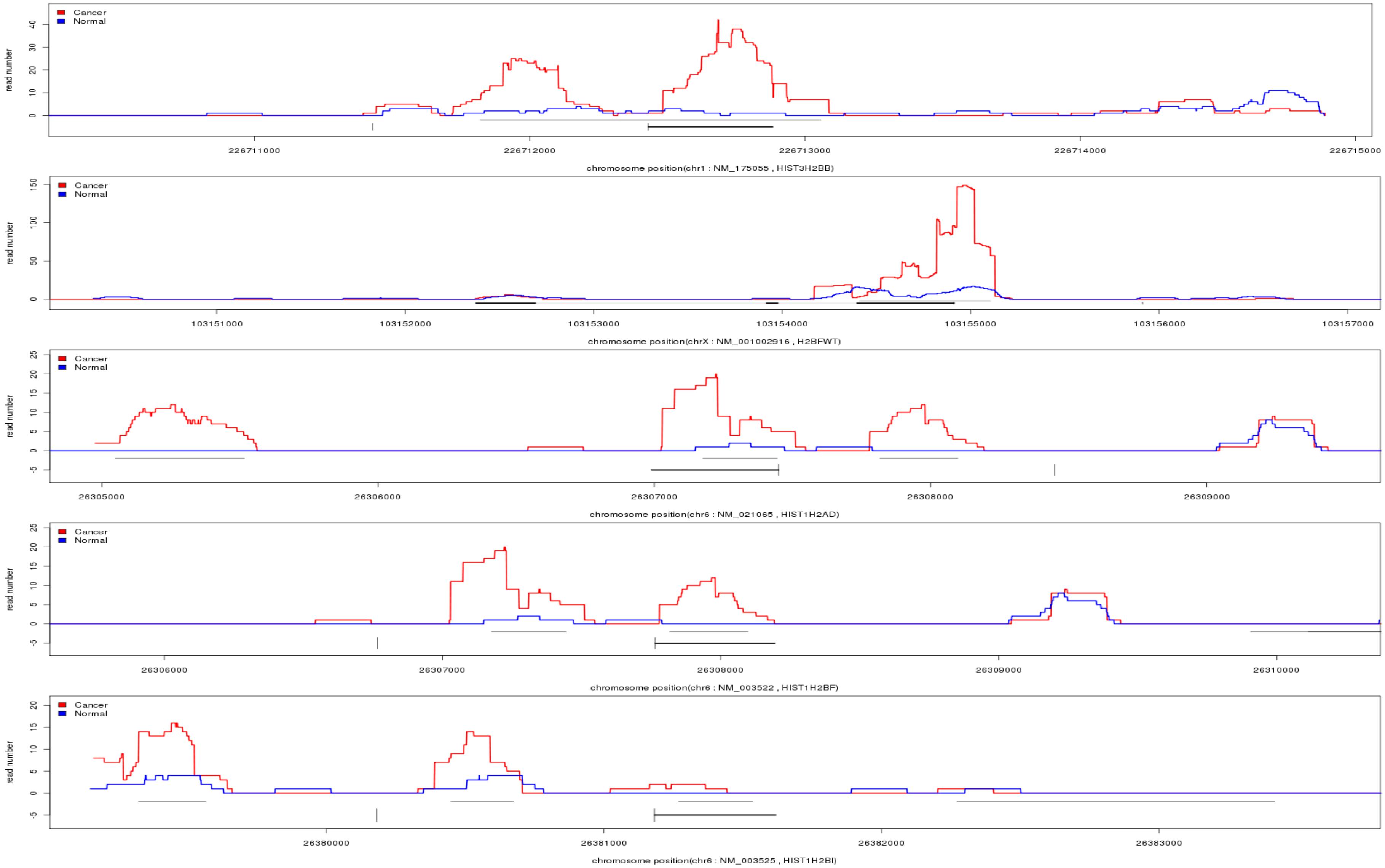
Cancer
Normal
read number
226711000
226712000
226713000
226714000
226715000
chromosome position(chr1 : NM_175055 , HIST3H2BB)
Cancer
Normal
read number
103151000
103152000
103153000
103154000
103155000
103156000
103157000
chromosome position(chrX : NM_001002916 , H2BFWT)
Cancer
Normal
read number
26305000
26306000
26307000
26308000
26309000
chromosome position(chr6 : NM_021065 , HIST1H2AD)
Cancer
Normal
read number
26306000
26307000
26308000
26309000
26310000
chromosome position(chr6 : NM_003522 , HIST1H2BF)
Cancer
Normal
read number
26380000
26381000
26382000
26383000
chromosome position(chr6 : NM_003525 , HIST1H2BI)

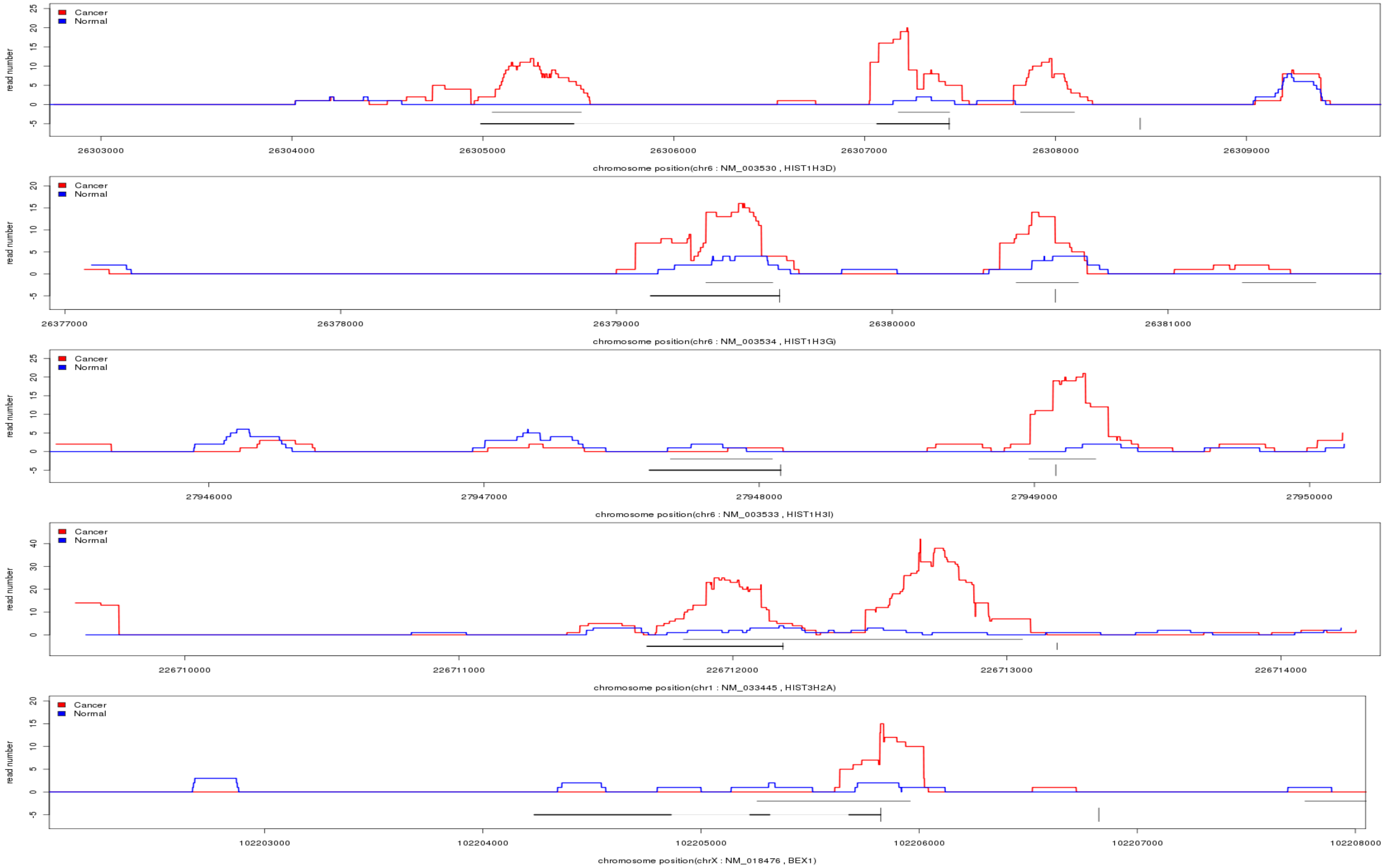

Plots of several genes with 5'CGIs hypermethylation. Red; cancer, blue; normal, gray; CG island, black; exon

Supplementary Fig. 9

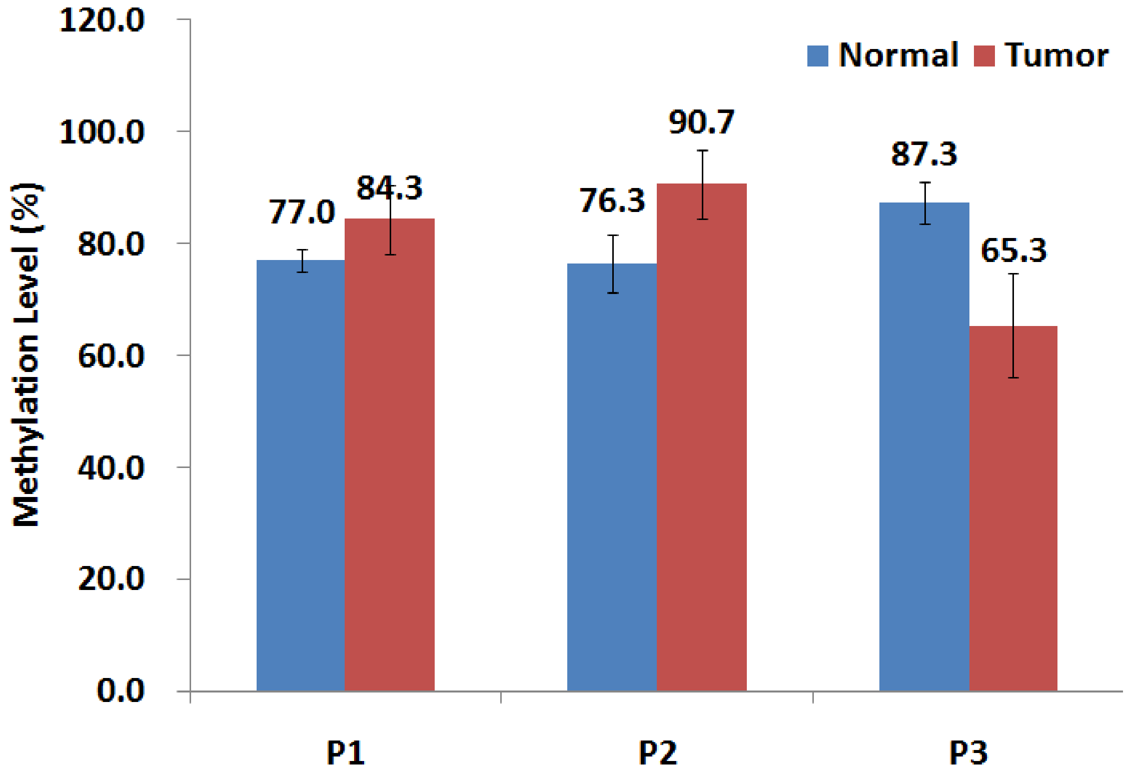

MDM2 methylation level of normal and cancerous tissue in three individual samples by pyrosequencing.

Supplementary Fig. 10

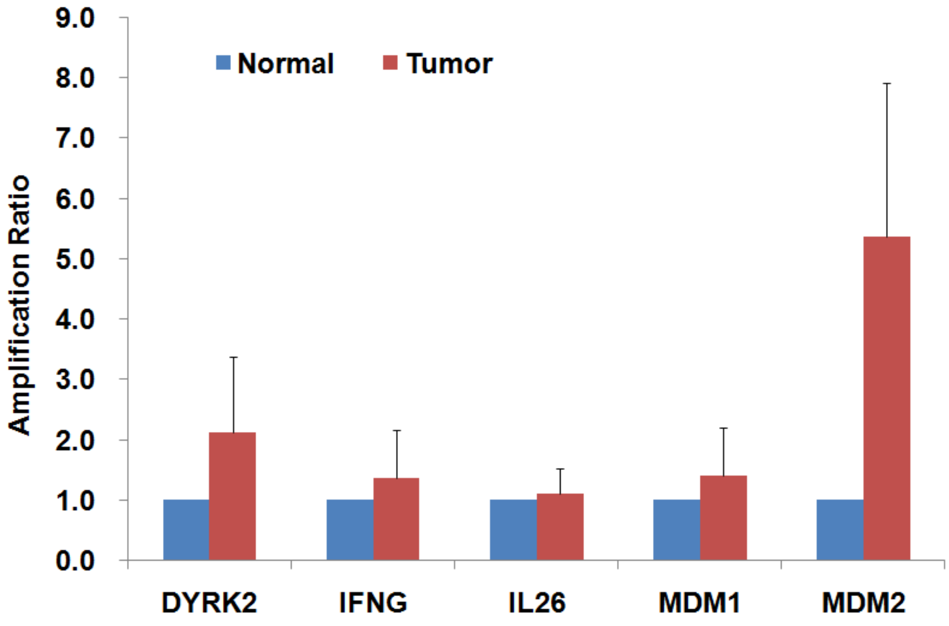

Amplification ratio of several genes (*DYRK2*, *IFNG*, *IL26*, *MDM1*, and *MDM2*) within chromosome 12q14 LRES region by real-time qPCR.

Supplementary Fig. 11

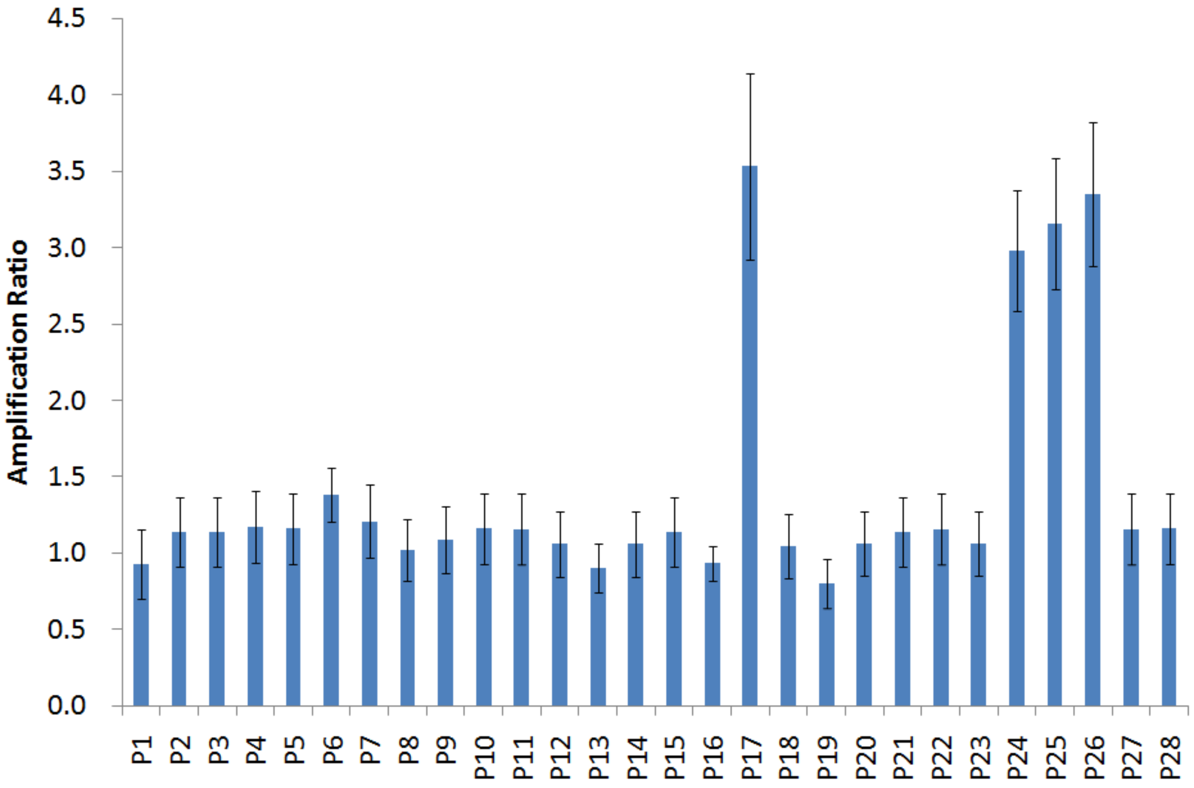

Amplification ratio of specific locus in an upstream region of *MDM2* by real-time qPCR.
